# Correlation Between An Email Based Board Review Program and American Board of Pediatrics General Pediatrics Certifying Examination Scores

**DOI:** 10.3885/meo.2009.Res00321

**Published:** 2009-11-09

**Authors:** Erik E. Langenau, Joshua Fogel, Henry A. Schaeffer

**Affiliations:** * Maimonides Infants and Children's Hospital of Brooklyn, Brooklyn, NY, USA; † Department of Economics, Brooklyn College of the City University of New York, Brooklyn, NY, USA

**Keywords:** Pediatrics, Board Review, Board Certification Exam, Email

## Abstract

**Objective:**

To investigate the impact of a weekly email based board review course on individual resident performance on the American Board of Pediatrics (ABP) General Pediatrics Certifying Examination for pediatric residents and, specifically, residents with low ABP In-training Examination (ITE) scores.

**Methods:**

Weekly board-type questions were emailed to all pediatric residents from 2004–2007. Responses to board-type questions were tracked, recorded, and correlated with ITE scores and ABP General Pediatrics Certifying Examination Scores.

**Results:**

With regard to total number of questions answered, only total number of questions answered correctly had a significant positive correlation with standard board scores (n = 71, r = 0.24, p = 0.047). For “at risk” residents with ITE scores ≤ 200 (n = 21), number of questions answered in PL 3 year (r = 0.51, p = 0.018) and number of questions answered correctly for all PL years (r = 0.59, p = 0.005) had significant positive correlations with standard board scores.

**Conclusions:**

Participating regularly in the email-based board review course, answering board style questions, and answering correctly to board style questions were associated with higher standard board scores. This benefit existed for all but was especially prominent among those with poor in-training examination scores.

For pediatric board certification, graduates from ACGME-sponsored pediatric residency programs are required to pass the American Board of Pediatrics (ABP) General Pediatrics Certifying Examination.^1^ Individual and aggregate board examination performance is used by many stakeholders: physicians, state licensing boards, third-party payers, employers, hospital credentialing committees, and the Pediatric Residency Review Committee (RRC). ABP Certification Examination performance has been positively correlated with USMLE Step 1 scores^2^ and ABP In-training Examination (ITE) scores.^3^

Although clinical experience and residency program-specific educational activities provide the knowledge base for residents as they prepare for board certification examinations, residents also use additional tools for board preparation. Among others, these methods may include board review books, courses, and study groups. In general, the educational value of commercial test preparation courses in clinical medicine has not been demonstrated.^4^ Specific educational interventions have been directed toward residents who were identified as “at risk” for poor performance on certification examinations in family medicine^5^ and surgery^6^–^9^ residency programs. Examination performance improvement strategies included individualized learning programs,^6^,^7^ weekly reading assignments,^5^,^6^,^8^,^9^ test-taking strategy review,^5^,^7^ content review,^5^–^7^ and practice questions.^5^–^9^

The purpose of this study is to investigate the effectiveness of the “eBoard Review” course — an innovative course that combines the use of board-type review questions, e-mail delivery of questions to individual residents, and weekly responses with feedback and teaching points. We hypothesize that participation will be positively correlated with ABP General Pediatrics Certifying Examination performance for both residents in general and residents “at risk” for poor performance on the board exam. As the predictive validity of the ABP ITE has been demonstrated in previous studies,^3^ risk stratification will be based on past performance on the ABP ITE. While previous studies have investigated educational interventions in relation to in-training examinations,^5^–^9^ there appears to be very limited research addressing board examination performance as an outcome measure. To our knowledge, there have been no studies addressing the effectiveness of board preparation on ABP Certifying Examination performance for pediatric residents.

## Methods

**“eBoard Review” Course Description** – For academic years 2004–5, 2005–6 and 2006–7, all pediatric residents at Maimonides Infants and Children's Hospital of Brooklyn were invited to voluntarily participate in a weekly electronic board review (“eBoard Review”) series, coordinated by the Associate Residency Program Director for Pediatrics. This program was developed for the purpose of promoting educational dialog between residents and improving pediatric certification examination performance.

**Figure 1.**
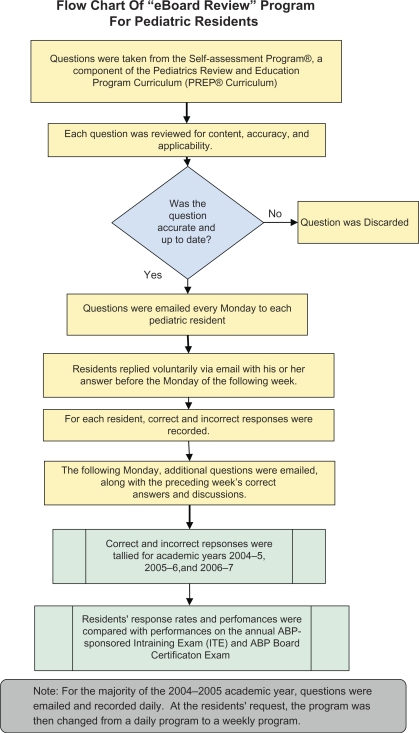


In this weekly “eBoard Review” program, residents received sample board questions by email. These questions were taken from the Self-assessment Program®, which is a component of the Pediatrics Review and Education Program Curriculum (PREP® Curriculum).^10^ As a residency benefit for pediatric residents at Maimonides Infants and Children's Hospital of Brooklyn, each resident receives a subscription to PREP® The Curriculum®, allowing access to the program's educational materials (such as sample board review questions from the Self-assessment Program®). Before distributing the questions via email, each question was reviewed for content, accuracy, and applicability. Questions were emailed weekly to each pediatric resident. Each resident then replied via email with his or her answer within the week, and responses were recorded. During the subsequent week, additional questions were emailed to each resident along with the preceding week's correct answers, discussions, and teaching points. Essentially, the program functioned as a weekly board review course administered throughout the academic year. A summary of the “eBoard Review” program is shown in Figure 1.

**Study Design** – From July 2004 to June 2007, all 2005, 2006 and 2007 graduating pediatric residents (N = 90) at Maimonides Infants and Children's Hospital of Brooklyn were invited to voluntarily participate in this study. During the 2004–5 academic year, 269 questions were distributed; during the 2005–6 academic year 358 questions were distributed; and during the 2006–7 academic year 266 questions were distributed. Unique questions were used each year, and residents did not receive duplicate questions during the study period. Data were collected from each of the 90 residents. Data included demographic characteristics, “eBoard Review” participation and performance indices, American Board of Pediatrics Annual In-training Examination (ITE) scores, and American Board of Pediatrics General Pediatric Certifying Examination scores. Table 1 summarizes the data collection methodology. The hospital's Institutional Review Board approval was received to analyze these data.

**Table 1.** Summary of Data Collection Methodology

Data	Mechanism of Data Collection
Resident Demographics	Resident Portfolio and ERAS application
“eBoard Review” participation/performance	Weekly record of responses
ABP In-training Examination (ITE)	ABP Report to Program Director
ABP General Pediatrics Certifying Examination	ABP Report to Program Director

Note: ABP = American Board of Pediatrics, ITE = In-training Examination, ERAS = Electronic Residency Application Service.

**Figure 2.**
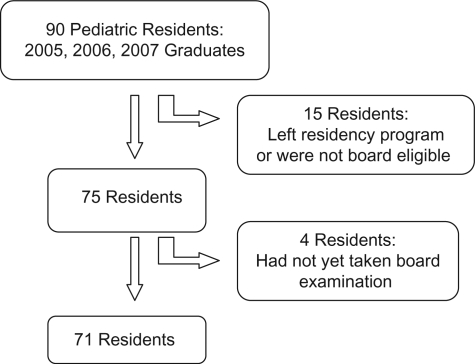


**Statistical Analysis** – Descriptive statistics were used to describe the demographic characteristics. For the continuous variables, Spearman correlation values were used for all the correlation analyses due to the small sample size. The Mann-Whitney test for skewed variables was used to analyze a number of continuous outcomes (number questions answered, number questions answered correctly) for the categorical independent variable of pass/fail on board scores. All p-values are two-sided. SPSS Version 16.02 (SPSS, 2008) was used for all analyses. For the outcome evaluation of certification exam performance, a flowchart of inclusion and exclusion criteria are shown in Figure 2. Of the original 90 residents, 15 left the program prematurely due to a variety of circumstances such as transfer to other programs, failure to meet program requirements for promotion, change in career; consequently, these residents either were not eligible to take the board certification examination or results were not reported back to the program director by the American Board of Pediatrics. In addition, 4 residents, who were board eligible and completed their full training, had not yet taken the board certification examination for a variety of personal reasons. Therefore, 71 residents completed the ABP certifying examination and were eligible for study analysis.

## Results

As shown in Table 2, the average age of participants was almost 33 years old. One-third attended a US medical school, one-quarter consisted of US citizens who attended schools outside of the US, and a little more than one-fifth had a DO degree.

**Table 2.** Demographic Characteristics of Pediatrics Residents Participating in “eBoard Review” (N = 90)

Variable	Mean	Percentage	Frequency
Age	32.89 (SD = 5.12)		
Gender			
Female		53.3%	48
Male		46.7%	42
US Medical School			
No		63.3%	57
Yes		33.7%	33
US Citizen Offshore School			
No		75.6%	68
Yes		25.4%	22
Physician Degree			
MD		78.9%	71
DO		21.1%	19
Previous Pediatric Postgraduate Training in Foreign Country			
No		84.4%	76
Yes		15.6%	14
Medicine-Pediatrics Resident			
No		95.6%	86
Yes		4.4%	4

Note: SD = Standard Deviation, MD = Doctor of Medicine, DO = Doctor of Osteopathic Medicine, Age is based on 71 individuals (those who took the American Board of Pediatrics Board Certification Examination).

Table 3 shows the descriptive statistics for questions answered and questions correctly answered. Although all residents (n = 90) elected to participate in the “eBoard Review” program, individual response rates varied significantly. Of the 90 residents, 16 (7 who were eligible for the study and 9 who were ineligible for the study) did not respond at all during the study period. With regard to questions answered by postgraduate level year (PL year), the greatest range was for PL3 year with a high of 324 questions, and the greatest mean value was for PL2 year at 69.73 questions. This pattern also existed for questions answered correctly with the greatest range for PL3 year with a high of 281 questions and the greatest mean value for PL2 year at 51.07 questions.

**Table 3.** Board Style Questions and Pediatrics Residents’ Response Rates (N = 90)

Variable	Residents	Low	High	Mean	SD
Number Questions Answered during PL Year					
PL1 year	26	0^*^	196	48.77	52.77
PL2 year	55	0^*^	312	69.73	75.99
PL3 year	76	0^*^	324	56.34	75.85
PL4 year	4	0^*^	80	39.75	32.72
Total number for PL1–PL4 years	90	0^*^	601	106.04	141.39
Number Questions Answered Correctly during PL year					
PL1 year	26	0^*^	143	33.77	39.36
PL2 year	55	0^*^	276	51.07	59.16
PL3 year	76	0^*^	281	42.51	60.77
PL4 year	4	0^*^	53	29.75	22.34
Total number for PL1–PL4 years	90	0^*^	514	78.19	109.56

Note: SD = Standard Deviation, PL= Postgraduate Level, PL4 = Med/Peds Residents.

Note: 269 questions were administered in 2004–5, 358 in 2005–6, and 266 in 2006–7.

Note: ^*^Although all residents elected to participate in the study, 7 of the eligible residents did not respond to review questions during the study period.

Table 4 shows Spearman correlation values of standard board scores with the board style questions. With regard to questions answered by PL year, both PL3 year and total number of questions approached significance for a positive correlation with standard board scores. With regard to questions answered correctly by PL year, PL3 year approached significance for a positive correlation, and total number of questions answered correctly had a significant positive correlation with standard board scores.

**Table 4.** Correlations of ABP Standard Board Scores with Board Style Questions (N = 71)

Variable	N	r	p-value
Number Questions Answered during PL Year			
PL1 year	16	−0.22	0.413
PL2 year	44	0.02	0.901
PL3 year	71	0.22	0.061
PL4 year	4	0.80	0.200
Total Number for PL1–PL4 years	71	0.22	0.065
Number Questions Answered Correctly during PL year			
PL1 year	16	−0.16	0.562
PL2 year	44	0.05	0.771
PL3 year	71	0.23	0.052
PL4 year	4	0.80	0.200
Total number for PL1–PL4 years	71	0.24	0.047

Note: PL= Postgraduate Level, PL4 = Med/Peds Residents

Only number questions answered for PL3 year (n = 71, r = 0.22, p = 0.067) and number of questions answered correctly for PL3 year (n = 71, r = 0.22, p = 0.060) had positive correlations approaching statistical significance with percentile board scores. No significant correlations were found between percentile board scores and questions answered or answered correctly based on total questions, questions in PL1 year or questions in PL2 year (data not shown).

Based on the Mann-Whitney test, pass/fail determination approached statistical significance for number questions answered for PL3 year (Fail: n = 24, M = 45.17, SD = 81.44; Pass: n = 47, M = 65.13, SD = 75.51; p = 0.075) and also number of questions answered correctly for PL3 year (Fail: n = 24, M = 34.75, SD = 68.07; Pass: n = 47, M = 48.83, SD = 59.22; p = 0.065) where those who passed had higher values for questions correct and questions answered correctly. There were no statistically significant differences for PL years, academic years, and totals for PL years in regards to pass/fail determination on board scores (data not shown).

For this particular residency training program, moving average ITE standard scores were 247.5 in 2004, 287 in 2005, and 318 in 2006. National moving average ITE standard scores for these years were 376, 368, and 362 respectively. Based on ITE performance, residents were stratified into risk groups; those with low ITE scores were identified as “at risk” for poor performance on the board certification examination. Based on the performance of the most recent ITE exam, risk levels were assigned to residents with ITE scores of ≤ 300, ≤ 250, and ≤ 200. As shown in Table 5, Spearman correlation analyses were conducted for “at-risk individuals” for the board style question variables for PL years that were either significant or approached significance with standard board scores. For those with ITE scores of <300, there were no significant correlations. For those with ITE scores of <250, number of questions answered in PL3 year, number of questions answered correctly in PL3 year, and total number of questions answered correctly for all PL years had significant positive correlations with standard board scores. Also, total number of questions answered for all PL years approached significance with a positive correlation with standard board scores. For those with ITE scores of <200, number of questions answered in PL3 year, number of questions answered for all PL years, total number of questions answered correctly in PL3 year, and total number of questions answered correctly for all PL years all had significant positive correlations with standard board scores. The highest correlations were seen within this subset of individuals with r values as high as 0.59.

**Table 5.** Correlations of ABP Standard Board Scores with Board Style Question Variables for PL Years (N = 71)

Variable	N	r	p-value
ITE≤300			
Number Questions Answered PL3 Year	40	0.26	0.109
Total Number Questions Answered for PL1–PL4 years	40	0.22	0.170
Number Questions Answered Correctly PL3 Year	40	0.25	0.115
Total Number Questions Answered Correctly for PL1–PL4 years	40	0.23	0.164
ITE≤250			
Number Questions Answered PL3 Year	29	0.38	0.040
Total Number Questions Answered for PL1–PL4 years	29	0.36	0.059
Number Questions Answered Correctly PL3 Year	29	0.42	0.023
Total Number Questions Answered Correctly for PL1–PL4 years	29	0.40	0.032
ITE≤200			
Number Questions Answered PL3 Year	21	0.51	0.018
Total Number Questions Answered for PL1–PL4 years	21	0.54	0.011
Number Questions Answered Correctly PL3 Year	21	0.56	0.008
Total Number Questions Answered Correctly for PL1–PL4 years	21	0.59	0.005

Note: ITE = In-training Examination, ITE represents most recent ITE score, PL = Postgraduate Level.

As stated above, 19 of the 90 residents were excluded from the study for leaving the residency program prematurely or not taking the ABP Certifying Exam at the time of analysis. These 19 did not differ significantly from the 71 included in the analyses with regard to gender (p = 0.56), US medical school attendance (p = 0.60), offshore medical school attendance by US citizen (p = 1.00), physician degree (p = 0.34), previous pediatrics training experience (p = 1.00), or Medicine-Pediatrics residency training (p = 0.58). Age was not analyzed, as age was measured for the age at the time of taking the boards and we did not have data on board status for those not included. With regard to risk groups, for those with ITE scores ≤ 300, the comparison did not significantly differ (p = 0.11). For those with ITE scores ≤ 250, there was a significantly (p = 0.004) greater percentage of those not included (78.9%, n = 15) than those included (40.8%, n = 29). For those with ITE scores ≤ 200, there was a significantly (p = 0.01) greater percentage of those not included (63.29%, n = 12) than those included (29.6%, n = 21).

## Discussion

Our study suggests that answering board style questions and answering correctly to board style questions are associated with higher standard board scores. This benefit exists for all, but is especially prominent among those with poor in-training examination scores. Our results also suggest that answering correctly, as opposed to simply answering, confers slightly additional benefits due to the greater significance levels and greater correlation values for answering correctly.

Two specific patterns were noted. Participation in the electronic board review (“eBoard Review”) course had medium to large significant correlations for “at-risk” residents with ITE scores of either ≤ 250 or ≤ 200. It is possible that either the increased knowledge acquired by completing these questions or participating in the program motivated them to study more on their own. Also, specifically for at-risk and PL3 residents, we noted significant patterns for a number of board score outcomes. Besides the motivation suggestion mentioned above, either the increased knowledge level at PL3 year confers the most benefit by answering board style questions or possibly increased interest by PL3 year due to upcoming board exams confers benefit. Therefore, residents who were nearing program completion (PL3) and who had low ITE scores might have been particularly motivated to prepare for the certifying examination, thereby taking advantage of every educational opportunity, including the “eBoard Review” program. As we did not have qualitative resident satisfaction survey data or formal measures of motivation, we can only speculate about the increased motivation of PL3 residents who are at risk for poor performance on the ABP Certifying Examination. Also, because the number of questions offered varied slightly between academic years, comparing the number of questions answered between PL years may not necessarily reflect motivational differences between the groups. A more formal study involving motivational measurement could be replicated in the future for all residents, PL3 residents and “at risk” residents.

Board-type review questions were disseminated by email so that all residents would be able to participate—residents located at different geographic clinical sites and residents with different on-call schedules. With three hospital-based sites, more than ten ambulatory sites, and more than twenty clinical rotations, scheduling educational activities for residents is particularly challenging. By using email, this educational program allowed for wide material dissemination and access to all residents, regardless of location and availability. Anecdotally, we found that residents often responded during nighttime hours while on-call, thereby maximizing their educational opportunities during nontraditional working hours. Previous studies have demonstrated that traditional and Internet-based CME programs are equally effective.^11^ Although we did not use an Internet-based program, we did use email to structure the “eBoard Review” program. This method of dissemination might have contributed to the impact of the board review program. Future studies could address resident satisfaction and comparing performance outcomes with methods of board-type question dissemination.

Because the predictive validity of the ABP ITE on future ABP General Pediatrics Certifying Examination performance has been established^3^, pediatric residency program directors can use the ITE scores to identify residents who are “at risk” for poor performance on the ABP Certifying Examination. Althouse and McGinnis presented five-year average passing rates for 2001–2005 ABP certification exam by ITE score groups, and extrapolating from their data, third year residents who score less than 200 on the ITE would have a 36.6% chance of passing the ABP Certification Examination^3^. To reflect the ITE reporting style used by the ABP and to reflect how the ITE scores are likely to be used by program directors, we also used overlapping risk group analysis. Developing educational programs specifically designed for these “at-risk” residents becomes very important for the residents themselves and their residency program directors. Residents with low ITE scores may be encouraged to participate in a board review course, such as the “eBoard Review” program, in efforts to improve their knowledge and exam performance.

Our study has a few limitations. First, the study was conducted at a single institution. To further evaluate the “eBoard Review” program's effectiveness and generalizability, the study should be duplicated at other training institutions. Second, residents with high ITE scores were likely to perform well on their ABP certification examination, regardless of whether they answered “eBoard Review” questions or not. This might account for the inability of this study to demonstrate a significant overall correlation between answering board-type questions and certification exam scores. Third, although all residents enrolled in the study, ongoing participation in the “eBoard Review” course was voluntary and self-directed. Therefore, consistent participation and individual response rates varied widely, and a significant number of residents did not respond at all during a particular academic year. Fourth, although all residents enrolled in the study and received these questions whether they responded electronically or not, a few residents reported anecdotally that they did read the questions and critiques on a regular basis but did not respond via email. This form of participation was not accounted for in our study and could potentially alter the study's overall results. Fifth, four residents had not yet taken the ABP Certification Examination at the completion of the study; this selection bias could potentially alter our study's findings. Sixth, a significant number of “at risk” residents with ITE scores less than 200 and 250 were excluded from the study. This can be explained by the fact that most of these 19 residents, who were excluded from the study, left the training program prematurely due to academic difficulties and were therefore more likely to have lower ITE scores. However, this selection bias could also potentially affect our study's findings.

As of September 2009, the “eBoard Review” course continues and is now coordinated by chief residents. Because of positive resident feedback and faculty recognition of the program as an effective tool for ABP board exam preparation, participation in the course is now mandatory. Data continues to be collected, and analysis is likely to be repeated in the future.

In conclusion, the above “eBoard Review” approach of sending board style questions by email appears to be useful to pediatric residents as preparation for their ABP General Pediatrics Certifying Examination. It is especially useful for those pediatric residents with poor in-training examination scores as they may have improved certification examination scores after completing such a program.

## References

[1] American Board of Medical Specialties How A Physician Becomes Board Certified [Web page].

[2] McCaskill QE, Kirk JJ, Barata DM, Wludyka PS, Zenni EA, Chiu TT (2007). USMLE step 1 scores as a significant predictor of future board passage in pediatrics. Ambulatory Pediatrics.

[3] Althouse LA, McGuinness GA (2008). The in-training examination: an analysis of its predictive value on performance on the general pediatrics certification examination. J Pediatr.

[4] McGaghie WC, Downing SM, Kubilius R (2004). What is the impact of commercial test preparation courses on medical examination performance?. Teach Learn Med.

[5] Shokar GS (2003). The effects of an educational intervention for “at-risk” residents to improve their scores on the In-training Exam. Fam Med.

[6] Kosir MA, Fuller L, Tyburski J, Berant L, Yu M (2008). The Kolb learning cycle in American Board of Surgery In-Training Exam remediation: the Accelerated Clinical Education in Surgery course. Am J Surg.

[7] Harthun NL, Schirmer BD, Sanfey H (2005). Remediation of low ABSITE scores. Curr Surg.

[8] de Virgilio C, Stabile BE (2005). Weekly reading assignments and examinations result in sustained improvement in American Board of Surgery In-Training Examination (ABSITE) scores. Am Surg.

[9] de Virgilio C, Stabile BE, Lewis RJ, Brayack C (2003). Significantly improved American Board of Surgery In-Training Examination scores associated with weekly assigned reading and preparatory examinations. Arch Surg.

[10] American Academy of Pediatrics PREP: Pediatric Review and Education Program [Website].

[11] Wutoh R, Boren SA, Balas EA (2004). eLearning: a review of Internet-based continuing medical education. J Contin Educ Health Prof.

